# Microstructural and microperimetric comparison of internal limiting membrane peeling and insertion in large idiopathic macular hole

**DOI:** 10.1186/s12886-023-03006-z

**Published:** 2023-06-14

**Authors:** Lingzi Liu, Zengyi Wang, Yanping Yu, Xiaohan Yang, Biying Qi, Ke Zhang, Wu Liu

**Affiliations:** 1 Beijing Tongren Eye Center, Beijing Tongren Hospital, Capital Medical University, No 1, Dongjiaominxiang, Dongcheng District, Beijing, 100730 China; 2 Beijing Ophthalmology and Visual Sciences Key Laboratory, Beijing, China

**Keywords:** Idiopathic macular hole, Pars plana vitrectomy, Internal limiting membrane, Optical coherence tomography, Microperimeter-3

## Abstract

**Background:**

The internal limiting membrane (ILM) insertion technique was widely used to treat large macular hole (MH) for the high closure rate. However, the prognosis of closed MH after ILM insertion compared to ILM peeling remains controversial. This study aimed to compare foveal microstructure and microperimeter in large idiopathic MH surgically closed by ILM peeling and ILM insertion.

**Methods:**

This retrospective, non-randomized, comparative study included patients with idiopathic MH (minimum diameter ≥ 650 μm) who underwent primary pars plana vitrectomy (PPV) with ILM peeling or ILM insertion. The initial closure rate was recorded. Patients with initially closed MHs were divided into two groups according to the surgery methods. The best-corrected visual acuity (BCVA), optical coherence tomography (OCT) and microperimeter-3 (MP-3) outcomes of two groups were compared at baseline, 1 and 4 months postoperatively.

**Results:**

For idiopathic MH (minimum diameter ≥ 650 μm), ILM insertion had a significantly higher initial closure rate than ILM peeling (71.19% vs. 97.62%, *P* = 0.001). Among 39 patients with initially closed MHs who were on regular follow-up, twenty-one were assigned to the ILM peeling group and 18 to the ILM insertion group. Postoperative BCVA improved significantly in both groups. The final BCVA (logMAR) (0.40 vs. 0.88, *P* < 0.001), macular hole sensitivity (19.66 dB vs. 14.14 dB, *P* < 0.001), peripheral sensitivity of macular hole (24.63 dB vs. 21.95 dB, *P* = 0.005), and fixation stability (FS) within 2 degrees (82.42% vs. 70.57%, *P* = 0.031) were significantly better and external limiting membrane (ELM) defect (330.14 μm vs. 788.28 μm, *P* < 0.001) and ellipsoid zone (EZ) defect (746.95 μm vs. 1105.11 μm, *P* = 0.010) were significantly smaller in the ILM peeling group than in the ILM insertion group.

**Conclusion:**

For initially closed MHs (minimum diameter ≥ 650 μm), both ILM peeling and ILM insertion significantly improved the microstructure and microperimeter in the fovea. However, ILM insertion was less efficient at microstructural and functional recovery after surgery.

**Supplementary Information:**

The online version contains supplementary material available at 10.1186/s12886-023-03006-z.

## Background

Over the past decade, the internal limiting membrane (ILM) insertion technique was widely used in treating macular hole (MH) larger than 400 μm for the high closure rate and good visual prognosis [1–3]. It could promote large MH healing by providing a scaffold for glial cells migration and photoreceptors rearrangement [1, 4, 5], stimulating the proliferation of glial cells [1, 5], offering neurotrophic factors [5], and sealing the hole to prevent the vitreous humor filling [6, 7]. However, the prognosis of closed MH after ILM insertion remains controversial [1, 8–12]. Some studies reported that ILM insertion improved MH closure rate, postoperative best-corrected visual acuity (BCVA), and structure of external limiting membrane (ELM) and ellipsoid zone (EZ) in eyes with MHs larger than 400 μm [1, 8]. In contrast, more articles expressed concerns about adverse effects of ILM insertion on postoperative recovery. Several studies compared ILM peeling and ILM insertion and indicated the poorer recovery of BCVA, ELZ, and EZ in the ILM insertion group [10–12]. It would take a longer time for the outer retinal structure to recover after ILM insertion [10].

The Manchester Large Macular Hole Study [13] and our previous study [14] both showed that the initial closure rate would decrease significantly after pars plana vitrectomy (PPV) combined ILM peeling when the diameter of MH is above 650 μm. Although these failure cases closed after the second surgery, the visual improvement was limited [15]. It seems necessary for large MH (minimum diameter ≥ 650 μm) to increase initial closure rate by applying ILM insertion.

At present, there are several comparative studies between ILM peeling and ILM insertion in the large MH, but few of them explored foveal microstructure, retinal function and their association after surgery. In addition, both closed and unclosed eyes were included in the prognostic analysis and comparison of the two surgical methods, which would mask the true surgical outcome to some extent [10, 16, 17]. Hence, this study included the initially closed MH (minimum diameter ≥ 650 μm) and compared foveal microstructure and function between ILM peeling and ILM insertion using optical coherence tomography (OCT) and microperimeter-3 (MP-3).

## Methods

Patients with idiopathic MH (minimum diameter ≥ 650 μm) who underwent 23-gauge PPV with ILM peeling or ILM insertion at Beijing Tongren Hospital from November 2016 to September 2020 were collected. The comprehension ophthalmological examinations were completed at baseline, 1 and 4 months after the operation, including Snellen BCVA, slit-lamp testing on the anterior segment and lens status, fundus photography (fundus camera, TRC-50; Topcon, Tokyo, Japan), OCT (Cirrus high-definition OCT; Carl Zeiss, Dublin, CA, USA) and MP-3 (microperimeter MP-3; NIDEK, Gamagori, Japan). The research was approved by the Medical Ethics Committee of Beijing Tongren Hospital, Capital Medical University and adhered to the tenets of Declaration of Helsinki.

### Patient eligibility

Inclusion criteria: 1) Patients with idiopathic MHs (minimum diameter ≥ 650 μm); 2) MH was successfully closed after initial surgery; 3) follow-up longer than 4 months; 4) complete examination data before and after operation.

Exclusion criteria: 1) closed MHs with bare retinal pigment epithelium (RPE); 2) high myopic with diopter < -6.00 or axial length > 26.00 mm; 3) history of ocular trauma; 4) history of previous vitreoretinal surgery; 5) any other fundus diseases.

### Surgical techniques

All patients underwent standard 3-port PPV using 23-gauge technique under local anesthesia. If needed, phacoemulsification and intraocular lens (CT ASPHINA 509 M; Carl Zeiss Meditec Inc, Jena, Germany) implantation were performed. The posterior hyaloid separation was induced in cases without posterior vitreous detachment and the vitreous was cut to the peripheral vitreous base. Then the peripheral retinal was carefully inspected and photocoagulation was conducted if the degenerated or tearing area existed. In the ILM peeling group, the ILM was peeled off in a circular fashion for approximately 1–1.5 disc diameters from the hole without any dye. In the ILM insertion group, 0.25% indocyanine green (ICG: Dandong Yichuang Pharmaceutical Co, Ltd, Liaoning, China) was injected into the eye to stain the ILM for no more than 20 s. Then, the ILM was centripetally peeled for approximately 0.5–1 disc diameters around the hole, retaining a link with the MH margin. The ILM flap was subsequently trimmed to an appropriate size if necessary and inverted into the hole from all sides of MH. Afterwards, fresh autologous blood was injected to cover the ILM flap in accordance with intraoperative conditions. Finally, abundant fluid-air exchange was performed and sterilized air or C3F8 was reserved in the eye at the end of surgery. All the surgeries were completed by one experienced surgeon (W.L.). Patients were asked to keep a face-down position for 5–10 days.

### OCT examination

**Fig. 1:**
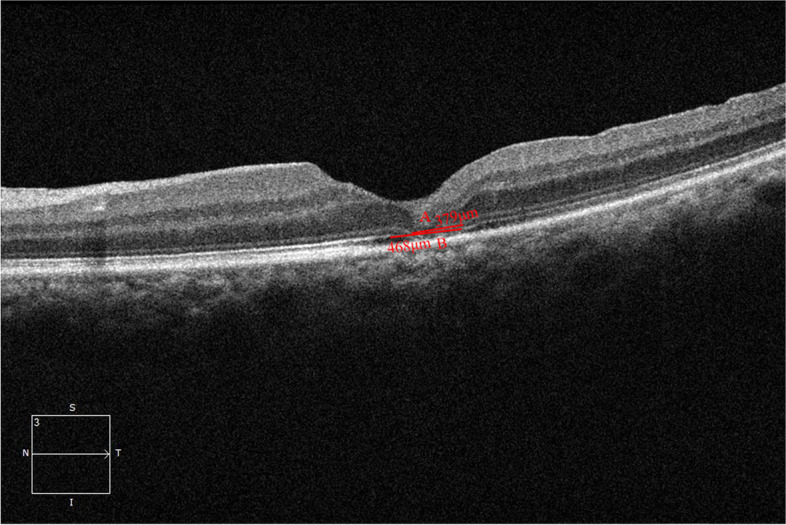
Measurements of the ELM defect (**A**) and EZ defect (**B**). The ELM defect and EZ defect were separately measured the narrowest line which roughly paralleled to the RPE between the edges of the broken ends of the neuroepithelia

The macular area was analyzed by horizontal high-definition 5-line raster scan and Macular Cube 512 × 128 scan pattern. The minimum diameter, ELM defect and EZ defect were separately measured the narrowest line which roughly paralleled to the RPE between the edges of the broken ends of the neuroepithelia, ELM and EZ on the largest horizontal cross section by using the OCT caliper function (Fig. 1). The closure status was determined by OCT at 1 month postoperatively. Holes with an RPE covered by retinal tissue or with high reflection material, that is, no RPE bare, were considered MH closed [2, 3, 18]. Two retinal specialists separately judged the status of glial proliferation in holes by OCT images.

### MP-3 examination

**Fig. 2:**
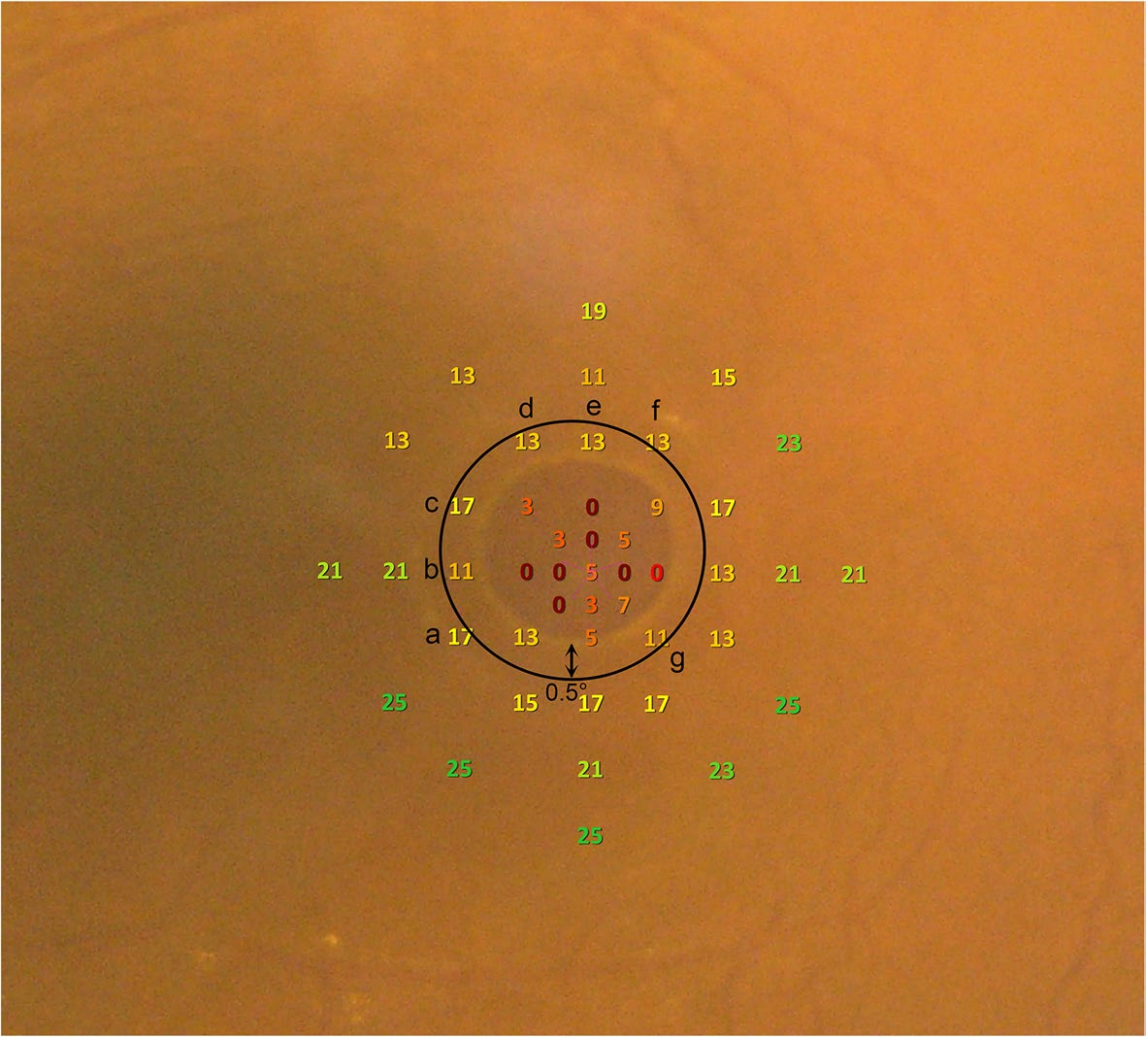
A Microperimeter-3 image of a 79-year-old female patient whose minimum diameter is 768 μm. Spot b (11 dB), c (17 dB), d (13 dB), e (13 dB), and g (11 dB) were included in the calculation of macular hole sensitivity. While spot a (17 dB) and f (13 dB) were excluded because their distance to macular hole margin was larger than 0.5 degree

MP-3 was used to evaluate retinal sensitivity and fixation stability (FS). The customized pattern consists of 45 stimulus loci within the 8-degree retina and the fixation target is shown as the 1-degree red circle. Goldmann III stimuli and a 4–2 staircase strategy were used. The range of stimulus luminance is 0–34 dB with maximum stimulus luminance of 10,000 asb. Apart from calculating mean macular sensitivity within 8 degrees, we manually calculated macular hole sensitivity [19], the average value of stimuli which scattered in the MH and 0.5 degree from the edge of MH (Fig. 2). In addition, we computed a valuable index, peripheral sensitivity of macular hole, which was the average value of stimuli outside the MH and within 1 degree from the edge of MH. This value was to evaluate the viability of retinal cells around the hole. Moreover, FS within 2 degrees was also measured.

### Statistical analysis

All the statistics were analyzed by SPSS software (SPSS for Windows, version 19.0, Chicago, IL, USA). Patients who met the criteria for inclusion were divided into two groups according to the surgery methods. Between two groups, Student’s t-test or Mann–Whitney U test was used for continuous variables and Fisher’s exact test for categorical variables. One-way repeated measures analysis of variance (ANOVA) or Friedman test followed by multiple pairwise comparisons using the Bonferroni adjustment method was performed to analyze the pre-and post-operative change. Spearman correlation analysis was performed for correlation analysis. *P* < 0.05 was considered statistically significant.

## Results

PPV with ILM peeling or ILM insertion was performed in 101 eyes of 101 patients with idiopathic MH (minimum diameter ≥ 650 μm). The initial closure rate was 71.19% (42 / 59) in patients who were treated with ILM peeling and 97.62% (41 / 42) in patients who were treated with ILM insertion. The initial closure rate in the ILM insertion group was significantly higher than in the ILM peeling group (*P* = 0.001). Of the 17 patients with persistent holes in the ILM peeling group, twelve patients underwent a second surgery where expanded ILM peeling and massage of the hole margin were conducted, while the remaining 5 patients refused reoperation. All the 12 patients obtained Type I closure. One patient in the ILM insertion group did not receive reoperation. Surprisingly, MH achieved Type I closure one year and seven months after the initial surgery. In the ILM peeling group, sterilized air was used to fill all the eyes. In the ILM insertion group, sterilized air and C3F8 were used to fill 36 eyes and 6 eyes, respectively. Patients who were further analyzed for recovery of the foveal microstructure and microperimeter were all filled with sterilized air.

Among these initially closed MH, thirty-nine eyes of 39 patients who were on regular follow-up and had complete examination date were included in this study. There were 21 eyes in the ILM peeling group and 18 eyes in the ILM insertion group. The baseline characteristics of the two groups are shown in Table 1. Baseline characteristics revealed no significant differences between the two groups.

**Table 1:** Baseline characteristics of patients with initially closed large idiopathic MHs

	ILM peeling (*n* = 21)	ILM insertion (*n* = 18)	*P*-value
Age (years)	63.10 ± 5.24	62.28 ± 11.26	0.780^a^
Sex (M / F)	3 / 18	1 / 17	0.609^b^
Eye (OS / OD)	12 / 9	12 / 6	0.742^b^
Eye axial (mm)	23.27 ± 1.12	23.47 ± 1.06	0.410^c^
BCVA (logMAR)	1.11 ± 0.36	1.21 ± 0.46	0.418^a^
MLD (μm)	738.14 ± 67.19	759.39 ± 95.53	0.422^a^
ELM defect (μm)	1463.62 ± 230.94	1588.44 ± 350.44	0.321^c^
EZ defect (μm)	1843.19 ± 361.84	1800.17 ± 319.57	0.698^a^
Mean macular sensitivity (dB)	16.51 ± 1.78	15.68 ± 3.03	0.318^a^
Macular hole sensitivity (dB)	7.29 ± 2.26	6.52 ± 3.09	0.375^a^
Peripheral sensitivity of macular hole (dB)	15.00 ± 3.26	13.28 ± 4.25	0.160^a^
FS within 2 degrees (%)	74.12 ± 18.40	65.86 ± 16.19	0.148^a^
Combined P + I (%)	90.48	66.67	0.112^b^

*BCVA* best-corrected visual acuity, *logMAR* logarithm of the minimum angle of resolution, *MLD* minimum linear diameter, *ELM* external limiting membrane, *EZ* ellipsoid zone, *FS* fixation stability, *P* phacoemulsification, *I* intraocular lens

^a^Student’s t test

^b^Fisher’s exact test

^c^Mann-Whitney test

The postoperative outcomes of the two groups are displayed in Table 2.

**Table 2:** Comparison of postoperative outcomes between two groups

	1 month			4 months		
	ILM peeling	ILM insertion	*P*-value	ILM peeling	ILM insertion	*P*-value
BCVA (logMAR)	0.59 ± 0.32	0.99 ± 0.45	0.003^a^*	0.40 ± 0.27	0.88 ± 0.43	0.001^b^*
ELM defect (μm)	605.81 ± 493.97	985.06 ± 367.53	0.012^b^*	330.14 ± 464.95	788.28 ± 379.01	0.001^b^*
EZ defect (μm)	1204.29 ± 370.02	1395.94 ± 337.08	0.101^a^	746.95 ± 441.24	1105.11 ± 365.21	0.010^a^*
Incidence of apparent glial proliferation (%)	-	-	-	23.81	61.11	0.025^c^*
Mean macular sensitivity (dB)	22.64 ± 2.31	19.79 ± 2.47	0.001^a^*	24.01 ± 2.16	21.30 ± 2.46	0.001^a^*
Macular hole sensitivity (dB)	16.88 ± 3.89	11.80 ± 4.05	0.001^a^*	19.66 ± 4.14	14.14 ± 4.20	0.001^a^*
Peripheral sensitivity of macular hole (dB)	23.12 ± 2.12	19.88 ± 4.29	0.008^a^*	24.63 ± 1.81	21.95 ± 3.53	0.005^b^*
FS within 2 degrees (%)	79.51 ± 15.33	65.33 ± 21.05	0.020^a^*	82.42 ± 11.05	70.57 ± 19.51	0.031^a^*

*BCVA* best-corrected visual acuity, *logMAR* logarithm of the minimum angle of resolution, *ELM* external limiting membrane, *EZ* ellipsoid zone, *FS* fixation stability

^*^P < 0.05

^a^Student’s t test

^b^Mann-Whitney test

^c^Fisher’s exact test

### Improvements in BCVA

The mean BCVA (logMAR) significantly improved at 1 month (*P* < 0.001, *P* = 0.002) and 4 months (*P* < 0.001, *P* < 0.001) postoperatively in both groups. The BCVA in the ILM insertion group was significantly poorer than that in the ILM peeling group at 1 month (0.59 ± 0.32 vs. 0.99 ± 0.45, *P* = 0.003) and 4 months (0.40 ± 0.27 vs. 0.88 ± 0.43, *P* < 0.001) postoperatively.

### Changes in OCT and comparison between apparent and non-apparent glial proliferation group

**Fig. 3:**
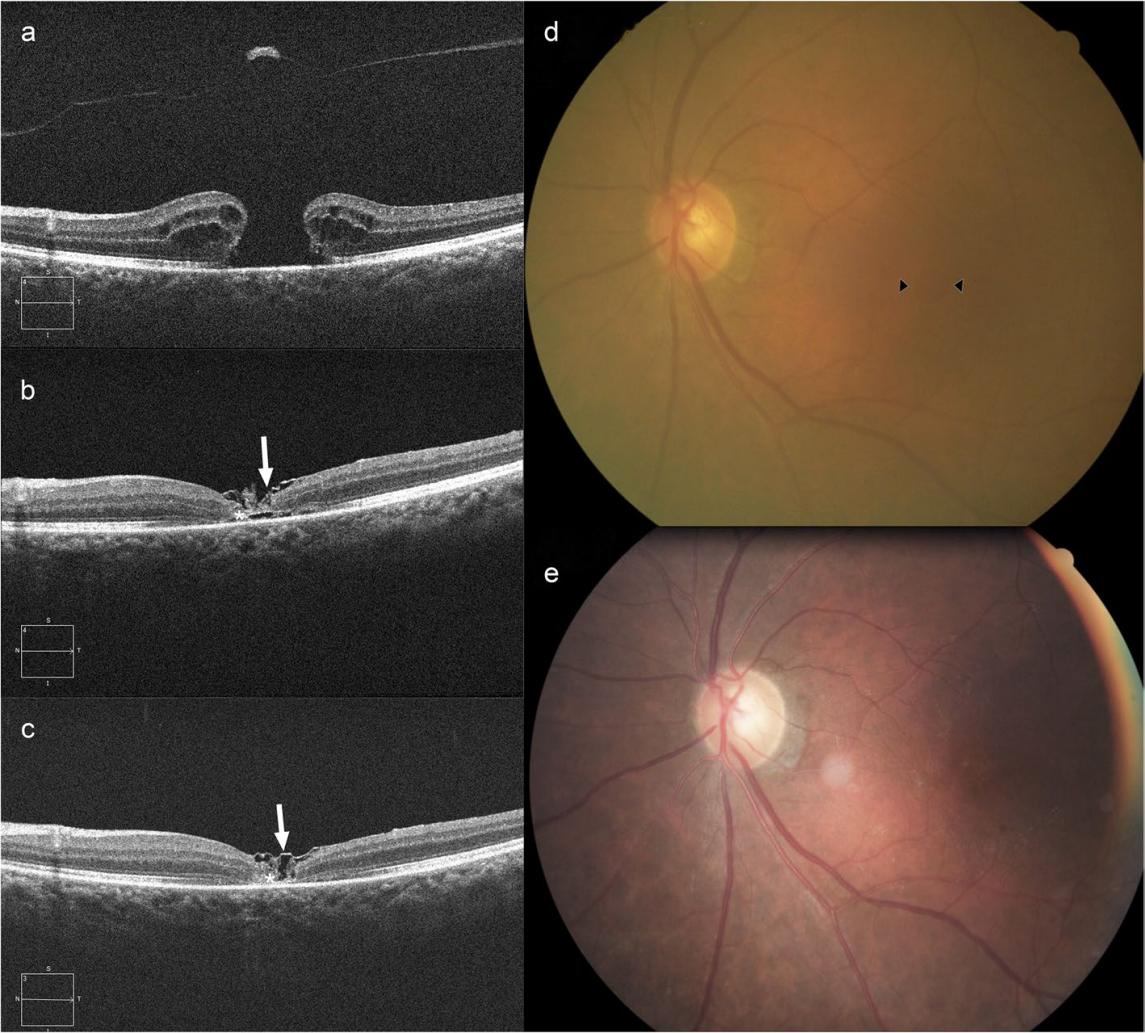
ILM insertion technique. **a** Preoperative OCT image shows a large MH with a diameter of 652 μm. BCVA and macular hole sensitivity are 1.3 logMAR and 4.70 dB before surgery. The preoperative sensitivity beyond MH (sensitivity of ILM peeling area) is 21 dB. **b** The 1-month postoperative OCT shows the MH closes with a mixture of multiple folded ILM flap (arrow) and glial tissue (asterisk). The ELM defect (1510 μm vs. 1224 μm) shortens while the EZ defect (1420 μm vs. 1915 μm) lengthens. BCVA remains 1.3 logMAR, macular hole sensitivity improves to 9.30 dB, and sensitivity beyond MH is 20.76 dB. **c** The 4-month postoperative OCT shows the tamponaded ILM flap (arrow) is still visible. A mass of glial tissue (asterisk) is in the hole, which inhibits the realignment of the ELM and EZ. BCVA remains 1.3 logMAR, macular hole sensitivity continuously improves to 13.40 dB, and sensitivity beyond MH is 21.60 dB. **d** The preoperative fundus photograph shows a MH. (arrowhead). **e** The 4-month postoperative fundus photograph shows the closure of the macular hole

**Fig. 4:**
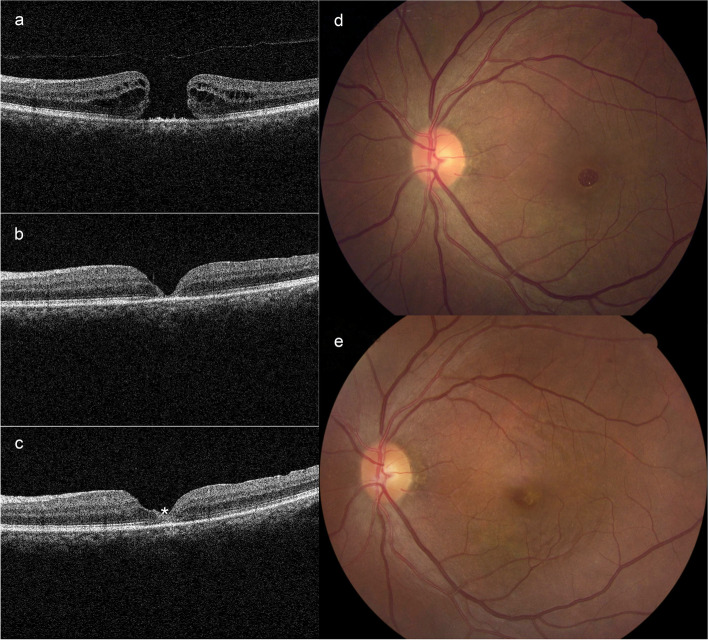
ILM peeling technique. **a** Preoperative OCT image shows a large MH with a diameter of 704 μm. BCVA is 1.52 logMAR and macular hole sensitivity is 7.20 dB before surgery. **b** The 1-month postoperative OCT shows MH closes without bare RPE. The ELZ (1530 μm vs. 631 μm) and EZ (1728 μm vs. 1338 μm) defects significantly shorten. BCVA and macular hole sensitivity improve to 1.00 logMAR and 10.90 dB. **c** The 4-month postoperative OCT shows the reflection of the ELZ and EZ is more distinctly visible. A small piece of glial tissue could be observed in the MH center (asterisk). The final BCVA is 0.22 logMAR and macular hole sensitivity is 12.50 dB. **d** The preoperative fundus photograph shows a MH. **e** The 4-month postoperative fundus photograph shows the closure of the macular hole

Significant decrease in ELM and EZ defects were observed in both groups at 1 month (ILM peeling group: *P* < 0.001, *P* < 0.001; ILM insertion group: *P* < 0.001, *P* = 0.001) and 4 months (ILM peeling group: *P* < 0.001, *P* < 0.001; ILM insertion group: *P* < 0.001, *P* < 0.001). ELM defect in the ILM peeling group was shorter than that in the ILM insertion group at 1 month (605.81 ± 493.97 μm vs. 985.06 ± 367.53 μm, *P* = 0.012) and 4 months (330.14 ± 464.95 μm vs. 788.28 ± 379.01 μm, *P* < 0.001) postoperatively, while the significant shorter EZ defect was only seen in the ILM peeling group at 4 months postoperatively (746.95 ± 441.24 μm vs. 1105.11 ± 365.21 μm, *P* = 0.010). The incidence of apparent glial proliferation was significantly different between the two groups (*P* = 0.025). Eleven of eighteen (61.11%) subjects in the ILM insertion group had a mass of high reflectivity materials in the fovea after 4-month surgery (Fig. 3). While in the ILM peeling group, five of twenty-one (23.81%) subjects had small pieces of high reflectivity materials in the fovea after 4-month surgery (Fig. 4). We divided cases into apparent glial proliferation group and non-apparent glial proliferation group. The comparison results demonstrated that BCVA (*P* = 0.003, *P* < 0.001), ELM defect (*P* < 0.001, *P* < 0.001) and peripheral sensitivity of macular hole (*P* = 0.035, *P* = 0.032) at 1 and 4 months after surgery was poorer in the apparent glial proliferation group, although these parameters before surgery showed no differences between the two groups.

### Changes in microperimetric parameters

**Fig. 5:**
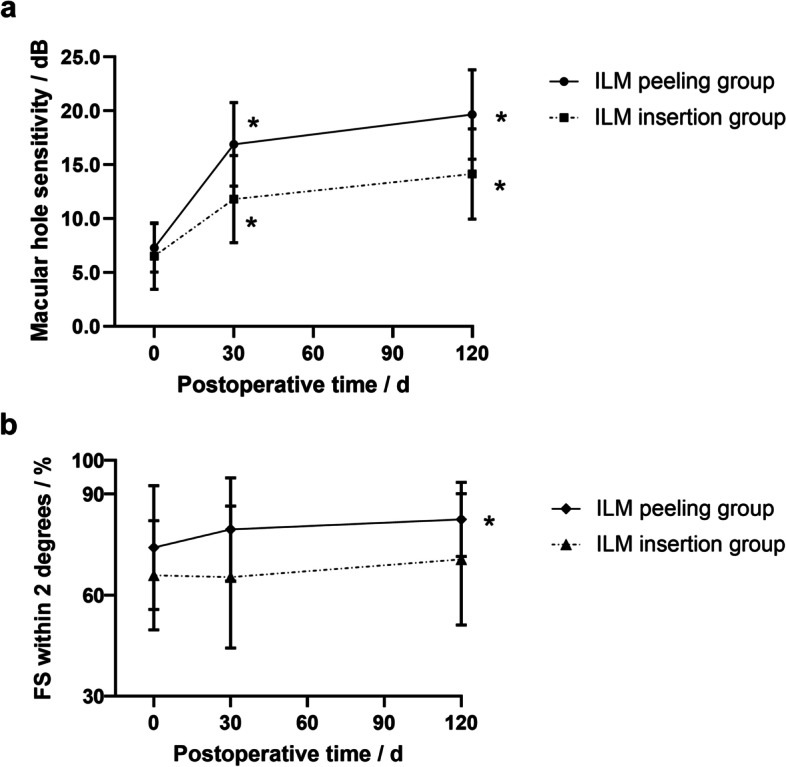
Line charts display the recovery process of macular hole sensitivity and FS within 2 degrees. **a** Macular hole sensitivity significantly elevates during the follow-up period in both groups. **b** In the ILM peeling group, FS within 2 degrees continuously improves, and significant improvement appears at 4-month after surgery. While in the ILM insertion group, FS within 2 degrees first declines at 1-month after surgery followed by a subsequent increase at 4-month after surgery. No significant difference is observed at each visit. *P < 0.05 compared to the preoperative status

The mean macular sensitivity increased at 1 month (*P* < 0.001, *P* < 0.001) and 4 months (*P* < 0.001, *P* < 0.001) in both groups. Significant differences were shown on mean macular sensitivity between the two groups at 1 month (22.64 ± 2.31 dB vs. 19.79 ± 2.47 dB, P < 0.001) and 4 months (24.01 ± 2.16 dB vs. 21.30 ± 2.46 dB, P < 0.001). The macular hole sensitivity increased at 1 month (*P* < 0.001, *P* < 0.001) and 4 months (*P* < 0.001, *P* < 0.001) in both groups. Significant differences were shown on macular hole sensitivity between the two groups at 1 month (16.88 ± 3.89 dB vs. 11.80 ± 4.05 dB, P < 0.001) and 4 months (19.66 ± 4.14 dB vs. 14.14 ± 4.20 dB, P < 0.001). The peripheral sensitivity of macular hole increased significantly at 1 month (*P* < 0.001, *P* < 0.001) and 4 months (*P* < 0.001, *P* < 0.001) in both groups. Significant differences were shown on peripheral sensitivity of macular hole between the two groups at 1 month (23.12 ± 2.12 dB vs. 19.88 ± 4.29 dB, *P* = 0.008) and at 4 months (24.63 ± 1.81 dB vs. 21.95 ± 3.53 dB, *P* = 0.005). In the ILM peeling group, FS within 2 degrees only significantly improved at 4 months (*P* = 0.013), while no significantly improved in the ILM insertion group in the follow-up period (Fig. 5). FS within 2 degrees in the ILM peeling group showed better recovery than the ILM insertion group at 1, 4 months (79.51 ± 15.33% vs. 65.33 ± 21.05%, *P* = 0.020; 82.42 ± 11.05% vs. 70.57 ± 19.51%, *P* = 0.031, respectively).

### Correlations of ELM and EZ defect with MP-3 results

Macular hole sensitivity (*r* = -0.725), mean macular sensitivity (*r* = -0.716), peripheral sensitivity of macular hole (*r* = -0.665), and FS within 2 degrees (*r* = -0.414) at 4-month postoperation were all significantly correlated with ELM defect at 4-month postoperation (*P* < 0.05), and significant correlations were also shown between macular hole sensitivity (*r* = -0.433), mean macular sensitivity (*r* = -0.418), peripheral sensitivity of macular hole (*r* = -0.617), and FS within 2 degrees (*r* = -0.514) at 4-month postoperation and EZ defect at 4-month postoperation (*P* < 0.05).

## Discussion

In this study, ILM insertion had a higher initial closure rate than ILM peeling (71.19% vs. 97.62%) in large MH (minimum diameter ≥ 650 μm). This result was comparable with previous studies [1–3] and suggested ILM insertion has high efficiency in the aspect of large MH closure. However, a closed MH does not mean a normal foveal microstructure and a good vision. Previously, several studies compared ILM peeling and ILM insertion and indicated the poorer recovery of BCVA, ELZ, and EZ in the ILM insertion group [10–12]. MP-3 could accurately measure the sensitivity of the corresponding points on the retina and assist in understanding the complex mechanism of deficiencies in ILM insertion technique [20–23]. Therefore, we combined OCT and MP-3 to explore the differences of microstructural and functional recovery in fovea between two ILM techniques. And we focused on those closed MH (minimum diameter ≥ 650 μm) to avoid error caused by closure rate.

Our results showed that BCVA in the ILM insertion group was significantly poorer than that in the ILM peeling group at 1 and 4 months postoperatively, although BCVA in both groups had significantly improved. We also found that the ELM and EZ defects were larger and macular hole sensitivity was inferior in the ILM insertion group at 1 and 4 months after surgery. Macular hole sensitivity was associated with ELZ defect and EZ defect (*r* = -0.725, *r* = -0.433), respectively. Interestingly, the incidence of high reflectivity materials which appeared in the foveal at 4 months after surgery (Fig. 3) in the ILM insertion group was far higher than the ILM peeling group (Fig. 4) (23.81% vs. 61.11%). These high-reflection materials were glial tissue [5, 24, 25]. These results indicated that ILM insertion affected the recovery of ELM and EZ negatively. It seemed that excessive glial proliferation occupied the original position of the photoreceptor and hindered their centripetal movement [26, 27]. Eventually, the density of photoreceptors in the fovea was relatively lower after ILM insertion [28], which manifested the larger ELM and EZ defects. Therefore, the macular hole sensitivity in the ILM insertion group was poor, as a sign of the poor recovery of the central vision. In this view, ILM insertion disrupted the self-repair of the photoreceptors. This might be associated with the different mechanisms between two techniques.

It was reported that glial proliferation also occurred early after the ILM peeling technique and the glial tissue would gradually reduce and be absorbed with time [29]. While we observed that there seemed no signs of disappearance of glial tissue in the ILM insertion group over time. Preceding studies supposed that the viability of the photoreceptors adjacent to MH influence retinal recovery [30]. Peripheral sensitivity of macular hole helps us know the function of photoreceptors adjacent to MH in detail by measuring the average sensitivity outside the MH and within 1 degree from the edge of MH. All cases were divided into apparent glial proliferation group and non-apparent glial proliferation group. We found peripheral sensitivity of macular hole at 1 and 4 months after surgery was poorer in the apparent glial proliferation group. Poorer peripheral sensitivity of macular hole indicated that the viability of the photoreceptors adjacent to MH was impaired. Some researchers consider that there is a balance between glial proliferation and photoreceptors realignment. If photoreceptors realignment is faster than glial proliferation (present as recovery of ELM integrity), it indicates that the postoperative BCVA is good. Otherwise, the glial tissue will fill the MH accompanied with photoreceptors defect [25, 31]. We speculated that application of ILM insertion in large MH broke this balance. Photoreceptors were difficult to move centrally and replace the excessive glial cells. Besides, the stimulation of ILM was sustained, so the glial tissue in the ILM insertion group scarcely reduced with time. In this condition, it was almost impossible to observe normal foveal microstructure in the ILM insertion group. Therefore, the impaired viability of the photoreceptors adjacent to MH may be another important reason for the poor BCVA in the ILM insertion group.

FS refers to the variability of fixation when people fixate intently on a stimulus over a certain period [32]. FS reflected visual quality and capability of capturing things rapidly and keeping watch for a long time. Patients who had bad FS complained that they had to spend longer time seeing the thing clearly despite visual acuity improvement [33]. FS could be quantitatively measured and expressed with FS within 2 degrees, which represents the percentage of fixation points located within a 2-degree circle for a certain time [34]. The higher values of FS within 2 degrees patients have, the more fixation stable they own. We noticed that FS within 2 degrees in the ILM insertion group did not significantly improve during the whole follow-up period, but it significantly improved at 4 months after operation in the ILM peeling group (Fig. 5). The reason probably was that a stable fixation could only be established on the basis of good function of photoreceptors. When the MH formed, the paracentral fixation would be established subsequently [35, 36], while during the healing of MH, the paracentral fixation would be broken, and the fixation would move toward the center gradually [37]. Then a new preferred retina location would be established somewhere in the original MH, where photoreceptors have relatively good functions [35]. Therefore, FS had a delayed recovery relative to the recovery of ELM, EZ and retinal sensitivity. In the ILM insertion group, the photoreceptors recovery seemed to be affected by the excessive glial proliferation, thus FS within 2 degrees needed a longer time to recover. This may be the reason why significant improvement of FS within 2 degrees could not be seen after ILM insertion in this study. We also found that FS within 2 degrees was negatively correlated with the ELM and EZ defects. However, FS recovery is a long process and varies among people. Although some patients’ ELM and EZ had recovered, their FS varied greatly. This demonstrated that ELM and EZ recovery were the basis not the representation of FS recovery. FS provides more information for doctors to evaluate the prognosis of ILM insertion.

In addition to whether or not the fovea is filled with ILM, the two surgical methods differ in the disposal of ILM, the application of autologous blood and dye. Compared to ILM peeling, the disposal of ILM is more complex and the risk of iatrogenic injury is higher in the ILM insertion. Therefore, it is essential to avoid the forceps touching the retina and to avoid inserting the ILM flap too deep to damage the RPE. The impact of autologous blood on the retina is not yet clear. Previous research speculated that toxicity from blood and fibrin degradation products may impair retinal tissue [38, 39]. However, the ILM flap serves as a barrier to mitigate the potential damage [39]. The impact of dye has been controversial [40–43]. The staining time was shortened as far as possible to reduce the chemical toxicity in this study. So far, no study of ILM insertion without dye has been reported, nor a comparative study of ILM insertion with or without dye. The impact of dye in ILM insertion can be further investigated.

ILM insertion technique indeed increased the surgical success of large MH and minimized the rate of reoperations. However, for those closed MHs (minimum diameter ≥ 650 μm), this study demonstrated that ILM insertion led to worse foveal structural and functional recovery. Therefore, it is worthwhile for surgeons to pay attention to the impact of the ILM insertion and the derived glial tissue after the operation. A more cautious attitude should be taken towards ILM insertion even in large idiopathic MH, and more advanced methods such as inverted ILM flap may be attempted for better outcomes [27].

Limitations of this study included retrospective, non-randomized study, relatively small sample size and short-term follow-ups. Consequently, a larger sample and longer period of prospective study are needed to evaluate the influence of ILM insertion on microstructure and microperimeter in the fovea. In conclusion, both ILM peeling and insertion significantly improved the foveal microstructure and microperimeter in initially closed MHs. However, ILM insertion was less efficient at microstructural and functional recovery after surgery.

## Supplementary Information

**Additional file 1.**

**Additional file 2.**

## Data Availability

All data generated or analysed during this study are included in this published article.

## Abbreviations

BCVA: Best-corrected visual acuity
ELM: External limiting membrane
EZ: Ellipsoid zone
FS: Fixation stability
ILM: Internal limiting membrane
MH: Macular hole
MP-3: Microperimeter-3
OCT: Optical coherence tomography
PPV: Pars plana vitrectomy
RPE: Retinal pigment epithelium
